# Cellular Fragments in the Perivitelline Space Are Not a Predictor of Expanded Blastocyst Quality

**DOI:** 10.3389/fcell.2020.616801

**Published:** 2021-01-05

**Authors:** Bo Yu, Helena T. A. van Tol, Tom A. E. Stout, Bernard A. J. Roelen

**Affiliations:** ^1^Farm Animal Health, Department of Population Health Sciences, Faculty of Veterinary Medicine, Utrecht University, Utrecht, Netherlands; ^2^Equine Sciences, Department Clinical Sciences, Faculty of Veterinary Medicine, Utrecht University, Utrecht, Netherlands; ^3^Embryology, Anatomy and Physiology, Department Clinical Sciences, Faculty of Veterinary Medicine, Utrecht University, Utrecht, Netherlands

**Keywords:** bovine, zona pellucida, cellular fragments, apoptosis, embryo quality, blastocyst

## Abstract

The presence of cellular fragments in the perivitelline space is a commonly used parameter to determine quality before transfer of *in vitro* produced (IVP) embryos. However, this parameter is difficult to assess after blastocyst expansion. In this study, we used mechanical hatching to confirm the presence of cellular fragments in the perivitelline space of bovine IVP blastocysts. We further looked for associations between possible apoptosis within extruded cells/ cellular fragments and the quality of bovine blastocysts using quantitative RT-PCR and immunofluorescence. Surprisingly, more than 42% of expanded blastocysts had cellular fragments in the perivitelline space; however, more than 37% of extruded cells were TUNEL negative. We observed no significant difference in embryo quality between expanded blastocysts with and without cellular fragments in the perivitelline space. Overall, our data suggest that embryos extrude abnormal cells to maintain their developmental potential. The presence of fragmented cells is not an indicator of embryo quality.

## Introduction

In the past 40 years, millions of human babies and bovine calves have been born with the help of *in vitro* fertilization (IVF) (Niederberger et al., 2018; Sirard, 2018). Despite these numbers, the clinical pregnancy rate after embryo transfer is relatively low, at around 30–40%. It has however been established that transfer of embryos with good gross morphology results in higher pregnancy rates (Lindner and Wright, 1983; Desai et al., 2000). Determination of embryo quality is challenging, however, because assessment of morphology is subjective and embryo grading results may vary between clinicians (Lindner and Wright, 1983; Farin et al., 1995; Baxter Bendus et al., 2006). In human IVF, it is common practice to transfer 4- to 8-cell stage embryos to the patient's uterus to shorten the *in vitro* embryo culture time. Currently, there is an increasing tendency to transfer at the blastocyst stage because this might improve implantation and clinical pregnancy rates (Papanikolaou et al., 2005; Gorodeckaja et al., 2019; Sciorio et al., 2019). Indeed, in cattle and most domestic animals, only transfer of morulae-blastocysts reliably leads to pregnancy (Van Soom et al., 2001). Therefore, a means to objectively assess the quality of blastocysts is of great importance for clinicians to aid selection of the best embryo to transfer.

There are two major approaches to evaluating blastocyst quality: invasive and non-invasive. Invasive evaluation of blastocyst quality mostly involves lysis of the embryo for gene expression analysis, fixation for imaging or preimplantation genetic testing (PGT). The total cell number and the ratio of inner cell mass (ICM) to trophectoderm cells are directly correlated with blastocyst quality (Leppens et al., 1996; Matsuura et al., 2010) but because the ICM is highly compacted, it is difficult to accurately determine either total or ICM cell number using regular microscopy. Thus, staining of the nuclei together with a marker for the ICM and/or trophectoderm lineage after fixation can be a more accurate way to obtain information on blastocyst quality in an experimental setting. Another valuable indicator of blastocyst health is the percentage of cells undergoing apoptosis, which is often assessed using the terminal deoxynucleotidyl transferase (TdT) mediated dUTP nick-end labeling (TUNEL) assay (Neuber et al., 2002). The disadvantage of these invasive evaluations of blastocyst quality is obvious, as these invasive procedures are not compatible with embryo survival. Therefore, in a clinical situation, embryo biopsy to remove a small number of cells for PGT is a more practical method to detect chromosomal or genetic abnormalities prior to embryo transfer, without the need to destroy the whole embryo. In commercial cattle breeding, analysis of cells to analyze for sex and assess genomic characteristics of the embryo is relatively common practice. However, due to extra costs, risks of biopsy and of misdiagnosis because of the difficulty of extrapolating data from a small number of cells to the whole embryo (e.g., in the case of chromosomal mosaics), or because of the limited number of genes that can be analyzed on PGT samples (Kalfoglou et al., 2005; Sermon et al., 2016; Cornelisse et al., 2020), non-invasive techniques based on gross morphological evaluation of embryos are still widely used in practice, particularly in human IVF (Lindner and Wright, 1983; Farin et al., 1995; Bormann et al., 2020).

An important parameter of non-invasive evaluation is the developmental stage in relation to time after fertilization. It has been shown that fast-cleaving embryos are of higher quality than slower-cleaving counterparts and expansion is considered to be an indicator of a “good” blastocyst (van Soom et al., 1997; Balaban et al., 2006). Another emerging non-invasive embryo selection technique is time-lapse embryo imaging, which allows continuous observation of embryo morphology without taking the embryo from optimal culturing conditions (Kovacs, 2014). However, this technique is not ready for routine clinical application, as clinically meaningful outcome parameters of time-lapse embryo imaging have not been fully determined yet (Armstrong et al., 2019).

One of the most commonly used parameters for assessing quality of blastocysts is the presence of extruded blastomeres and the extent of cellular fragmentation in the perivitelline space. Embryos with extensive cellular fragmentation are considered as “fair” or “poor” embryos likely to result in a low likelihood of implantation (Van Soom et al., 2003; Racowsky et al., 2010).

Once a blastocyst expands, the perivitelline space becomes very small, which makes it difficult to identify cellular fragments. Thus, there is a lack of information about cellular fragments in the perivitelline space at the blastocyst stage and its relationship to embryo quality.

To investigate the correlation of the presence of cell fragments in the perivitelline space with blastocyst quality, we mechanically hatched the embryo from the zona pellucida of expanded blastocysts. Surprisingly, there was no significant difference in the quality of blastocysts with or without cells or cellular fragments in the perivitelline space of day 8 blastocysts. Our data confirmed that embryos can tolerate a certain level of cell death and still reach the blastocyst stage. It is suggested that apoptosis and fragmentation are methods to remove abnormal cells from the embryo, and thereby protect the developmental competence of the embryo.

## Materials and Methods

### *In vitro* Embryo Production

All chemicals were purchased from Sigma Aldrich (St. Louis, MO, USA), unless otherwise stated. Cattle ovaries were obtained from a local slaughterhouse within 2 h after slaughter. Cumulus-oocyte complexes (COCs) were aspirated from 2-8 mm follicles using a winged infusion set (18G) connected to a vacuum aspiration system. COCs were isolated from the aspirated follicular fluid using a stereomicroscope. Groups of 40–60 oocytes were matured and fertilized *in vitro* as described previously (Brinkhof et al., 2015). In short, the oocytes were cultured in maturation medium: M199 (Life Technologies, Bleiswijk, The Netherlands) supplemented with 0.05 IU/mL recombinant hFSH (Organon, Oss, The Netherlands) and with 1% (v/v) penicillin-streptomycin (Life Technologies). Culture was for 23 h at 38.5°C, in an atmosphere of 5% CO_2_-in-air after which oocytes were fertilized by co-incubation with 1^*^10^6^/mL motile frozen/thawed sperm cells. The day on which COCs were co-incubated with sperm was considered as day 0 of embryo development. After incubation with sperm for 20–22 h, zygotes were denuded of their cumulus cells by vortexing for 3 min and then transferred to synthetic oviductal fluid (SOF) [107.63 mmol/L NaCl, 25 mmol/L NaHCO_3_, 7.16 mmol/L KCl, 1.19 mmol/L KH_2_PO_4_, 1.78 mmol/L CaCl_2_·2H_2_O, 3.20 mmol/L Sodium DL-lactate (60% syrup), 0.74 mmol/L MgSO_4_·7H_2_O (Merck Millipore, Billerica, MA, USA), 0.33 mmol/L Sodium pyruvate, 2.05 mmol/L L-Glutamine, 4 mg/mL BSA (Merck Millipore), 10 U/mL penicillin-streptomycin (Life Technologies), 1% MEM NEAA, 2% BME Amino Acids and 0.5 μL/mL Phenol Red 0.5% in LAL water (Lonza, Basel, Switzerland)] for further development at 38.5°C, in a humidified atmosphere containing 5% CO_2_ and 7% O_2_. On day 5, embryos were transferred to fresh SOF and cultured until day 9 (Brinkhof et al., 2017).

### Mechanical Separation

Day 7–9 expanded blastocysts were collected and placed in a washing medium composed of 6.66 mg/mL NaCl (Merck, Schiphol-Rijk, The Netherlands), 0.24 mg/mL KCl (Merck), 0.17 mg/mL NaHCO_3_, 0.05 mg/mLNaH_2_PO_4_ (Merck), 0.22% (v/v) of a 60% sodium lactate solution, 2.38 mg/mL HEPES, 0.20% (v/v) phenol red, 0.39 mg/mL CaCl_2_·2H_2_O, 0.10 mg/ mL MgCl_2_·6H_2_O (Merck), 0.11 mg/mL sodium pyruvate, 100 U/mL penicillin-Streptomycin (Gibco, Paisley, UK) and 6.00 mg/mL bovine serum albumin fraction V (BSA) (MP Biomedicals, Santa Ana, CA, USA), set at an osmolality of 280 ± 2 osmol/kg and pH 7.3 ± 0.05. Embryos were mechanically separated from their zonae pellucida using tungsten needles in washing medium under a stereomicroscope. Isolated embryos and zonae pellucidae were rinsed in PBS before collection for quantitative reverse transcription PCR and immunofluorescence.

### RNA Isolation and cDNA Generation

Groups of 47–55 zonae pellucidae containing obvious cell clumps or cellular fragments, and groups of 21–32 embryos were collected and stored in 100 μL RLT buffer (Qiagen, Venlo, The Netherlands) at −80°C until RNA isolation. Total RNA extraction and genomic DNA digestion was performed using the RNeasy Micro Kit (Qiagen) according to the manufacturer's instructions. Complementary DNA (cDNA) was synthesized directly after RNA extraction. The mixture used for reverse transcription (RT) was made up of 10 μL of the RNA sample and 4 μL of 5 × RT buffer (Invitrogen, Breda, The Netherlands) in a total volume of 20 μL containing 10 mM DTT (Invitrogen), 0.5 mM dNTP (Promega, Leiden, the Netherlands), 8 units RNAse inhibitor (Promega) and 150 units Superscript III reverse transcriptase (Invitrogen). Minus-RT controls were prepared from 5 μL RNA sample using the same reagents as above, without the reverse transcriptase. After incubation at 70°C for 5 min, the mixtures were cooled down on ice for 5 min, followed by 1 h at 50°C, and 5 min at 80°C. cDNA samples were stored at −20°C.

### Quantitative Reverse Transcription-PCR

Primer pairs (Eurogentec, Maastricht, the Netherlands) were designed on Primer-Blast (http://www.ncbi.nlm.nih.gov/tools/primer-blast) according to *Bos taurus* mRNA (Genbank; http://www.ncbi.nlm.nih.gov/nucleotide). The quantitative reverse transcription PCR (qRT-PCR) mixture contained 10 μL iQ SYBR Green supermix (Bio-Rad, CA, USA), 9 μL RNAse-free and DNAse-free water (Invitrogen) and 1 μL cDNA with a final primer concentration of 500 nM. The specificity and annealing temperature of primers (Supplementary Table 1) were first established using a temperature gradient from 55 to 68°C, and cDNA from 100 blastocysts as template. Reactions were performed on the CFX detection system (Bio-Rad) based on the manufacturer's protocol. Mixtures were kept at 95°C for 3 min, followed by 40 cycles of denaturation at 95°C for 20 s, the primer specific annealing temperature for 20 s and extension at 72°C for 20 s. To verify the purity of the amplified products, melting curves were generated after amplification with temperature increments of 0.5°C from 65 to 95°C for 5 s each step. To calculate amplification efficiency, standard curves of primers were generated by 4-fold dilutions of cDNA from 100 blastocysts in each reaction. All the reactions were performed on three independent biological cDNA samples in duplicate. Expression of *RPL15, SDHA*, and *YWHAZ* was used for normalization (Brinkhof et al., 2015).

### Immunofluorescence

Blastocysts and zonae pellucidae with cellular fragments were collected and fixed in 4% paraformaldehyde (PFA) for 15 min and then stored in 1% PFA at 4°C until further use. After washing twice in PBST (1x PBS, 0.01% Triton X-100), samples were blocked in PBS with 5% goat serum and 0.3% Triton X-100 for 60 min. Following three washes in PBST, samples were incubated with mouse monoclonal antibody against CDX2 (Biogenex, CA, USA; CDX2-88; 1:200) and Alexa Fluor™ 568 Phalloidin (Invitrogen; A12380; 1:100) in dilution buffer (1x PBS, 1% BSA, 0.3% Triton X-100) overnight. After three washes in PBST, secondary antibody incubation was performed with goat anti mouse Alexa488 (Invitrogen) for at least 1 h in the dark. Subsequently, nuclei were stained with DAPI (Sigma Aldrich) for 20 min in the dark. After three washes in PBST, samples were mounted with Vectashield (Brunschwig Chemie, Amsterdam, The Netherlands) on slides and stored at 4°C in the dark before imaging. Fluorescent images were obtained using a confocal laser scanning microscope (SPE-II-DMI4000; Leica, Son, The Netherlands) with a Z-stack projection and were further analyzed using IMARIS software (Bitplane, Zürich, Switzerland).

### Combined TUNEL and Immunofluorescence

Apoptotic cells were identified by terminal deoxynucleotidyl transferase dUTP nick end labeling (TUNEL) based on the Click-iT Plus TUNEL assay (Thermo Fisher Scientific, Carlsbad, CA, USA) with a few modifications. After fixation, samples were washed twice in PBST and incubated with 100 μL of TdT reaction buffer at 37°C for 10 min, followed by incubation with TdT reaction mixture for 60 min at 37°C. After two washes in Milli Q water, samples were incubated with 3% BSA and 0.1% Triton X-100 in PBS for 5 min at room temperature. Following two washes in PBST, samples were incubated with 100 μL of the Click-iT Plus TUNEL reaction cocktail for 30 min at 37°C in the dark.

After three washes in PBST, immunofluorescence and imaging were performed as described above.

### Statistical Analysis

Statistical analysis was performed using Excel and GraphPad Prism 8 (https://www.graphpad.com/scientific-software/prism/). Pools of embryos or zonae with cellular fragments from three biological replicates were analyzed for gene expression. Individual embryos or zonae with cellular fragments from three biological replicates were analyzed after immunostaining. Differences between two groups were analyzed by two-tailed unpaired Student's *t-*tests, and differences between multiple groups were examined by one-way ANOVA, followed by a *post-hoc* Tukey test. A linear mixed model [SPSS (IBM, Amsterdam, Netherlands)] was used to investigate differences in the total cell number, CDX2 positive cell number and TUNEL positive cell number per blastocyst and cellular fragment comparison. The “individual embryo” was set as “subject,” different parameters as “fixed effect” and “run of IVF” as “random effect” because of the variability between batches of ovaries. Statistical significance was set at *P* < 0.05.

## Results

### The Presence of Cellular Fragments in the Perivitelline Space of Expanded Blastocysts

**Figure 1:**
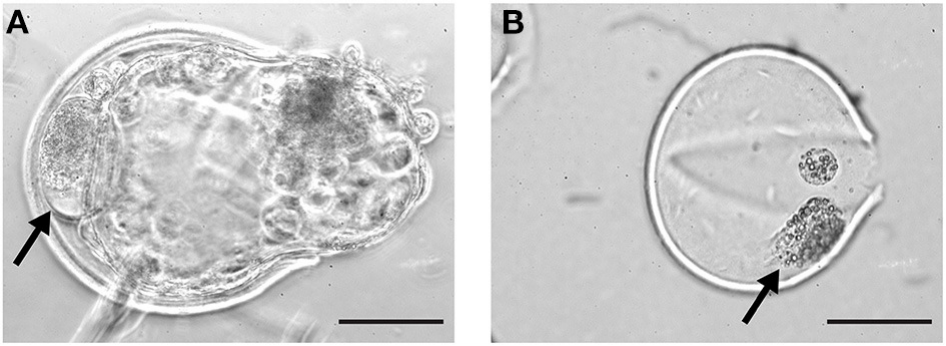
Cellular fragments in day 9 bovine blastocysts. Fragmented cells (arrows) in the perivitelline space of a day 9 hatching blastocyst **(A)** and within the zona pellucida of a hatched blastocyst **(B)**. Scale bar = 50 μm.

**Figure 2:**
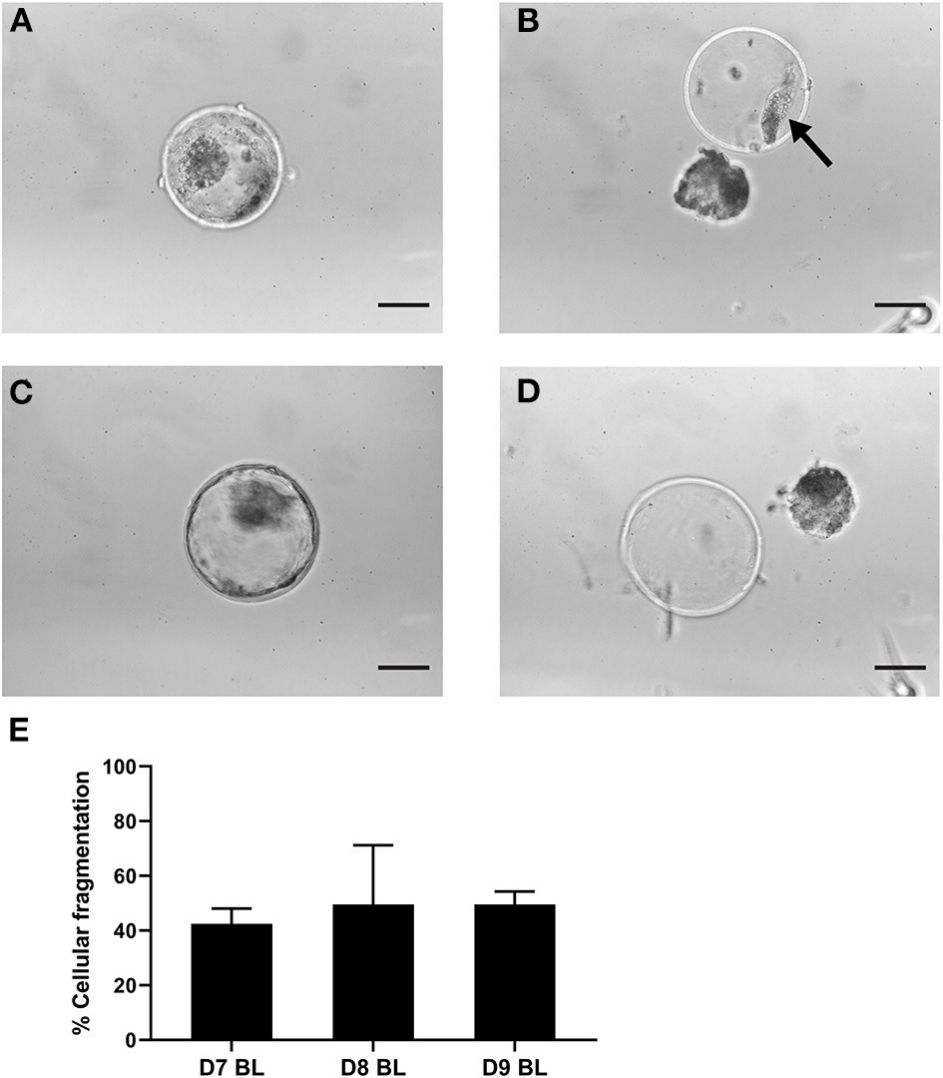
Cellular fragments in the zona pellucida of expanded blastocysts. Representative expanded blastocyst with cellular fragmentation before **(A)** and after **(B)** mechanically induced hatching. Representative expanded blastocyst without cellular fragments before **(C)** and after **(D)** mechanically induced hatching. Arrow indicates cell fragments. **(E)** Graph indicating percentages of blastocysts with cellular fragments in the zona pellucida after 7 (*n* = 140), 8 (*n* = 155), 9 (*n* = 148) days of *in vitro* culture. Error bars indicate standard deviation of three biological replicates. Scale bar = 50 μm. D, day; BL, blastocyst.

As cellular fragments can be detected in the perivitelline space of day 9 hatching and hatched blastocysts (Figure 1), we presumed that fragmented cells would also be present in the zonae of expanded blastocysts but would not be visible due to the pressure of the expanding blastocyst against the zona pellucida. To preserve the integrity of blastocysts and zonae pellucidae, we examined the presence of cellular fragments following manual removal of the zona pellucida of day 7 (*n* = 140), 8 (*n* = 155) and 9 (*n* = 148) blastocysts (Supplementary Table 2). As expected, fragmented cells were also detected between the embryo and the zona pellucida of expanded blastocysts (Figures 2A,B). Note that the occurrence of fragmented cells in the perivitelline space is difficult to establish before mechanical separation (Figures 2A–D). To our surprise, more than 42% of expanded blastocysts demonstrated cells or cell fragments in the perivitelline space with no significant difference between days 7, 8, and 9 blastocysts (Figure 2E, Supplementary Table 2).

### The Level of Apoptosis in Cellular Fragments

**Figure 3:**
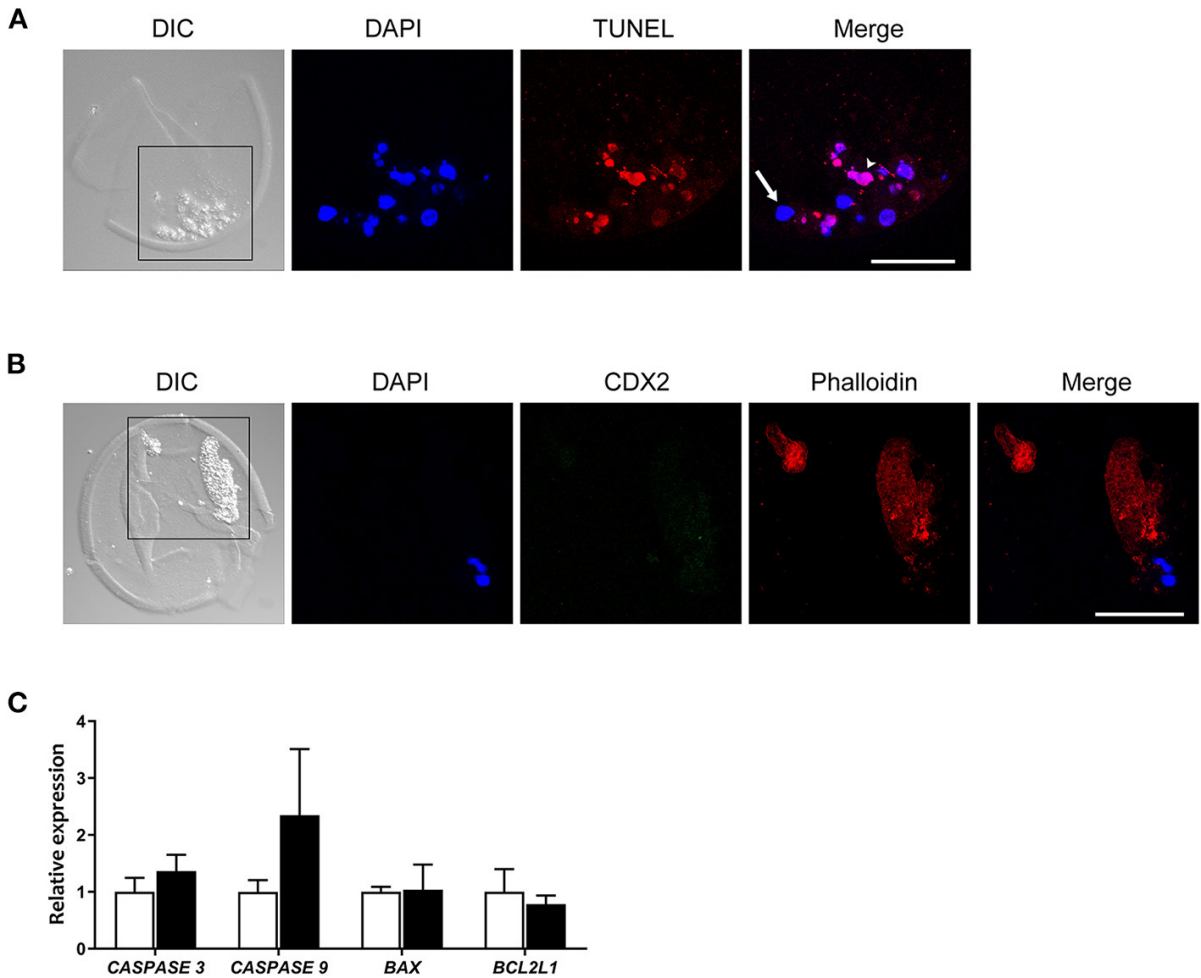
CDX2 and Phalloidin immunofluorescent staining, and apoptosis related gene expression in cellular fragments in the perivitelline space of a day 9 bovine blastocyst. **(A)** TUNEL positive (red, arrowhead) and negative cells (arrow) are indicated in a day 9 blastocyst. Blue represents DAPI staining. **(B)** CDX2 and Phalloidin staining of cellular fragments in a day 9 blastocyst. Black boxes in **(A,B)** indicate the areas presented in the right at higher magnification. Scale bar = 50 μm. **(C)** Relative expression of apoptosis-related genes -in day 9 blastocysts (white bars, *n* = 87) vs. fragmented cells (black bars, *n* = 151) as determined by qRT-PCR. Error bars indicate standard deviation of three biological replicates. DIC, differential interference contrast.

It has been suggested that cellular fragmentation in embryos is the result of elimination of abnormal cells (e.g., with chromosomal errors), and is closely related to apoptotic processes (Haouzi and Hamamah, 2009; Daughtry et al., 2019). We first used DAPI to examine nuclear DNA in fragmented cells in the zona pellucida of day 9 blastocysts, with 16/25 (64%) containing DAPI positive fragments. We then evaluated the level of possible apoptosis-related changes in the cells and cellular fragments in 11 zonae of day 9 blastocysts by means of the TUNEL assay. TUNEL positive cells were detected in the majority of cell clumps. Interestingly, we noticed that a mean of 37.4% of the extruded cells were TUNEL negative and contained morphologically normal nuclei as observed after DAPI staining (Figure 3A, Supplementary Table 3), indicating that a significant number of cells were extruded before cell death was initiated.

Since the cytoskeletal architecture plays a role as sensor and mediator of apoptosis (Desouza et al., 2012), we reasoned the actin cytoskeleton presents in cellular fragments. We therefore analyzed the occurrence of actin cytoskeletal elements using phalloidin staining. We observed large phalloidin positive areas in 10 fragmented cells of day 9 blastocysts (Figure 3A, Supplementary Table 3), which further suggested that fragmented cells were undergoing apoptotic processes. To examine whether the fragmented cells originated from the trophectoderm, cells were immunostained for CDX2 expression together with phalloidin. No CDX2 expression was detected in fragmented cells, including fragmented cells with an intact nucleus (Figure 3B). Together, these data suggest that either the cells were extruded by embryos before the differentiation of trophectoderm, or that trophectoderm cells rapidly lost CDX2 expression after exclusion from the embryo.

To evaluate the difference in apoptosis-related pathways between day 9 blastocysts (*n* = 87) and extruded cells (*n* = 151), we next performed qRT-PCR to determine expression of genes in the caspase and Bcl-2 families (Brentnall et al., 2013). Expression of *RPL15, SDHA*, and *YWHAZ* was used as a reference for quantification, and expression of these genes followed similar patterns in samples of day 9 blastocysts and extruded cells (data not shown). In contrast to expectations, the expression of caspase family genes was not statistically different between extruded cells and blastocysts (*P* > 0.17 for *CASPASE 3* expression and *P* > 0.11 for *CASPASE 9* expression). In addition, the expression levels of *BAX* (pro-apoptotic regulator) and *BCL2L1* (anti-apoptotic regulator) were similar in blastocysts and extruded cells (Figure 3C).

### Blastocyst Quality Comparison

In order to determine whether the presence of cells in the perivitelline space is an indicator of reduced blastocyst quality, we compared the quality of day 8 blastocysts with and without cellular fragmentation using invasive methods.

**Figure 4:**
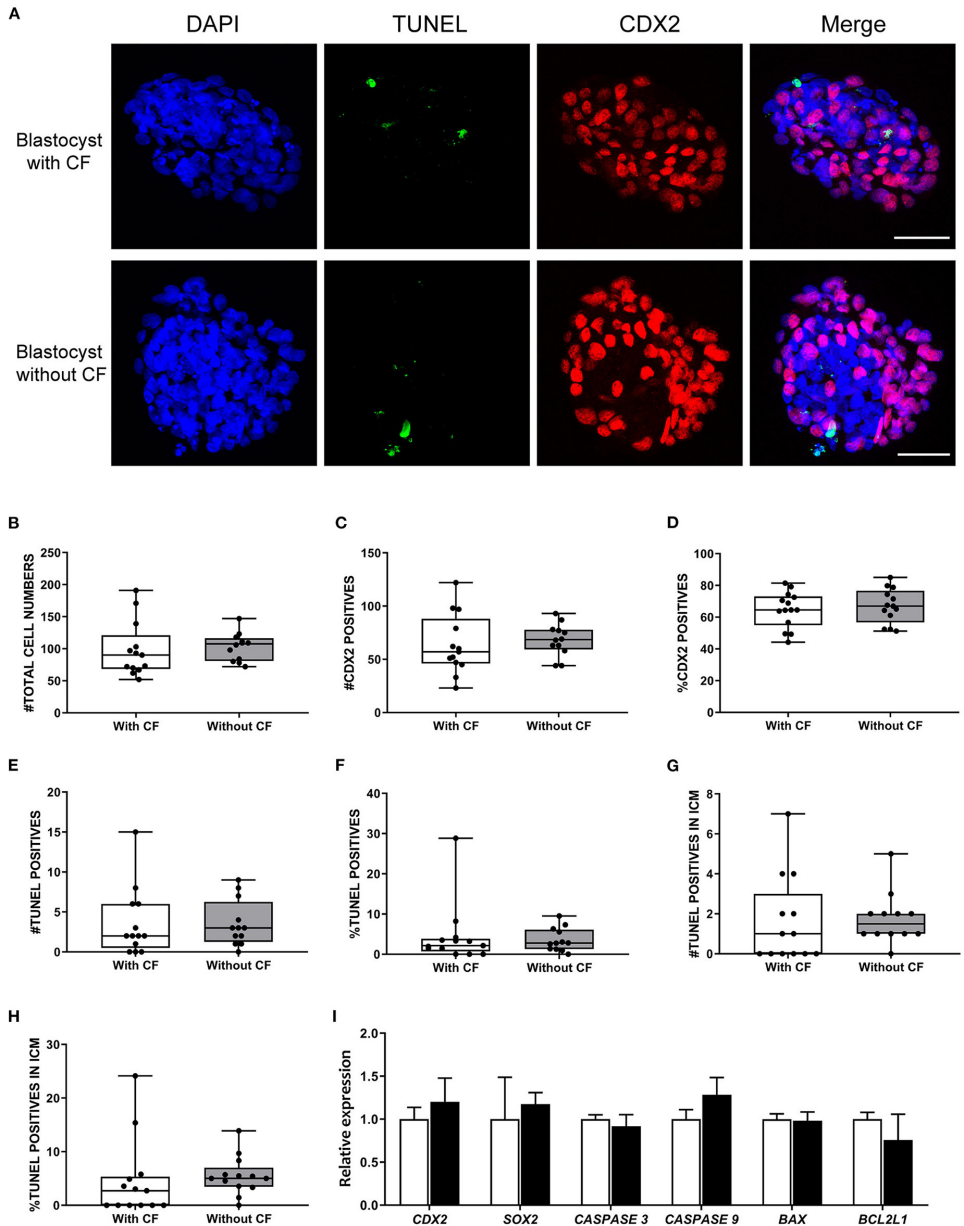
Frequency of apoptosis and ICM to trophectoderm ratio comparison between expanded bovine blastocysts with and without cellular fragments. **(A)** TUNEL (green) and CDX2 (red) immunofluorescence of day 8 blastocysts with and without cellular fragments. Scale bar = 50 μm. Box-whisker plots of total cell number **(B)**, CDX2 positive cells **(C)**, percentage of CDX2 positive cells **(D)**, total TUNEL positive cell number **(E)**, percentage of TUNEL positive cells **(F)**, TUNEL positive cell number in inner cell mass (ICM) **(G)**, percentage of TUNEL positive cell number in ICM **(H)** in blastocysts with cellular fragments (white, *n* = 64) or in blastocyst without (gray, *n* = 66) fragmented cells in the perivitelline space. **(I)** Relative expression of genes in day 8 blastocysts with (white bars) or without (black bars) fragmented cells, as determined by qRT-PCR. Error bars indicate standard deviation of three biological replicates. BL, blastocyst; CF, cellular fragmentation.

To simultaneously investigate cell death and the ratio of ICM to trophectoderm cells at the single blastocyst level (*n* = 25), we performed TUNEL staining combined with immunofluorescence staining for CDX2 (trophectoderm biomarker) and DAPI staining to label all nuclei (Figure 4A). The percentages of trophectoderm cells were similar between blastocysts with and without cells/cellular fragments in the perivitelline space, 64.6 and 67.0% CDX2 positive cells, respectively (Figures 4B–D). A small but similar proportion of TUNEL positive cells was detected in blastocysts with (6.5%) and without (4.5%) fragmented cells (Figures 4E,F). TUNEL positive cells were also detected in presumptive ICM cells (CDX2 negative; Supplementary Tables 4, 5). Nevertheless, a similar and low percentage of TUNEL positive cells was found in presumptive ICM cells in blastocysts with (4.6%) and without (5.5%) cellular fragmentation (Figures 4G,H).

We further examined the expression of *CDX2* and *SOX2* (ICM biomarker) and apoptosis regulator genes in day 8 blastocysts. In agreement with the TUNEL/combined immunofluorescence results, there were no significant differences in the levels of *CDX2, SOX2, CASPASE 3, CASPASE 9, BAX*, and *BCL2L1* expression between blastocysts with (*n* = 64) and without (*n* = 66) cellular fragmentation (Figure 4I).

## Discussion

Despite being used in clinical practice for decades, embryo evaluation prior to transfer is a challenging and subjective procedure. The presence of cellular fragments in the perivitelline space can be identified by clinicians, and is an important empirical criterion of embryo quality (Van Soom et al., 2003). However, our data indicate that the presence of extruded or fragmented cells is common in expanded blastocysts and is not a reliable indicator of blastocyst quality.

Although cellular fragments can be often observed in the perivitelline space of cleavage stage embryos, their presence in the same location around expanded blastocysts is less easy to confirm by regular stereomicroscopy. To identify their presence in expanded blastocysts, we mechanically separated zonae pellucidae and embryos, which allowed examination of the cells detached from the embryo and attached to the zona pellucida. Using electron microscopy, several groups have previously identified fragmented cells or “debris” in the perivitelline space (Vajta et al., 1997; Fair et al., 2001; Rizos et al., 2002), but further examination of the cells was not possible.

Recently, it has been reported that cell fragments contain chromosomal material, possibly resulting from encapsulated micronuclei (Daughtry et al., 2019). Indeed, we detected that more than half of cell fragments were DAPI positive and most of these DAPI positive cells were positive for TUNEL in day 9 blastocysts; nevertheless, we still found more than one third of cell fragments to be TUNEL negative.

In bovine embryos, cellular fragmentation has only been observed after the 8-cell stage, typically from the morula stage onwards (Gjorret et al., 2003; Van Soom et al., 2003). Similarly, apoptosis is also first observed after the 8-cell stage in bovine embryos (Byrne et al., 1999; Gjorret et al., 2003), coinciding with activation of the embryonic genome. Indeed, we found no CDX2 expression in the cellular fragments and there was no difference in the percentages of blastocysts with cellular fragments within the zona pellucida between days 7, 8, and 9 blastocysts, indicating that the cells were excluded from the embryo before the blastocyst stage. Possibly, embryos assess the genomic integrity of cells at around the time of embryonic genome activation and extrusion and fragmentation of cells with chromosomal errors is initiated at this time. Alternatively, the cellular fragments observed at the blastocyst stage result from the progressive degeneration of cells excluded prior to blastocyst formation. This may explain why the majority of cellular fragments were TUNEL positive, i.e., (programmed) cell death may have started after cell exclusion. Removal of cells by programmed cell death within the developing embryos would mainly affect the cell that later form the fetus, i.e., the ICM (Leidenfrost et al., 2011).

Interestingly, the detected mRNA levels in the cell fragments indicate that mRNA was not rapidly degraded. Indeed, the appearance of nuclear material within the fragments, as visualized using DAPI staining, suggested many nuclei were intact. In addition, the levels of caspase gene expression were not increased above those observed in the embryos themselves. These results indicate that not every extruded cell or cell fragment had undergone apoptosis, or that a portion of cellular fragments were at an early stage of apoptosis.

The technique of qRT-PCR does not allow determination of caspase protein activity, is partly dependent on post-translational modification (Parrish et al., 2013). Caspase mRNA can however be used as supplementary evidence of apoptosis (Krishnan et al., 2005; Zhang et al., 2019). Moreover, consistent with caspase gene expression, we did not find any significant differences in *BAX* (pro-apoptotic) and *BCL2L1* (anti-apoptotic) expression between day 9 blastocysts and day 9 zonae pellucidae with cellular fragments, or day 8 blastocysts with and without cellular fragments.

Generally, the presence of cellular fragments in the perivitelline space is considered as an indicator of poor embryo quality (Jurisicova et al., 1998; Chi et al., 2011; Maurer et al., 2015), although it has also been proposed that low levels of fragmentation have no negative impact on implantation and pregnancy rates (Alikani et al., 1999). In our study, there was no significant difference in embryo quality between day 8 blastocysts with cellular fragments and blastocysts without. Since in bovine IVF an average of only 30–40% of the embryos reach the blastocyst stage, it is possible that the embryos with high percentages of chromosomally abnormal cells were already arrested during early embryonic development, and the blastocysts we collected were of relatively high quality. Indeed, few TUNEL positive cell were detected in the blastocysts we collected.

To conclude, the presence of cellular fragments in the perivitelline space is common in expanded blastocysts. A limitation of this study is that even blastocysts with good morphology will not necessarily implant and give rise to healthy offspring. Blastocyst morphology is however a criterion used in practice to select or prioritize embryos for transfer. Although the most convincing demonstration of bovine blastocyst quality, i.e., birth of a healthy calf, has not been examined, we conclude that there is no correlation between the presence of cellular fragments in the perivitelline space and blastocyst quality.

## Data Availability Statement

The original contributions presented in the study are included in the article/Supplementary Material, further inquiries can be directed to the corresponding author/s.

## Author Contributions

BY, HT, TS, and BR conceived and designed the experiments. BY, HT, and BR performed the experiments and collected the data. BY analyzed the data. BR contributed the reagents, materials, and analysis tools. BY, TS, and BR wrote the manuscript. All authors read and approved the manuscript.

## Conflict of Interest

The authors declare that the research was conducted in the absence of any commercial or financial relationships that could be construed as a potential conflict of interest.
