# Supplementary material for: Cellular Fragments in the Perivitelline Space Are Not a Predictor of Expanded Blastocyst Quality
Source: Front Cell Dev Biol. 2021 Jan 5;8:616801. doi: 10.3389/fcell.2020.616801 (PMC7813684; doi:10.3389/fcell.2020.616801)
Supplement: Supplementary file 1 [file Data_Sheet_1.docx]

**Supplementary Table 1.** List of primers used for quantitative RT-PCR. Sense and anti-sense sequences are indicated by s and as respectively. Ta = annealing temperature.

| **Gene** | **NCBI** | **Sequence (5'-3')** | **Sense** | **Ta (°C)** |
| --- | --- | --- | --- | --- |
| *BAX* | NM_173894.1 | GCAGAGGATGATCGCAGCTG | s | 62 |
|  |  | CCAATGTCCAGCCCATGATG | as |  |
| *BCL2L1* | NM_001077486.2 | CGTGGAAAGCGTAGACAAGGAG | s | 62 |
|  |  | GTAGAGTTCCACAAAAGTGTC | as |  |
| *CASPASE3* | NM_001077840.1 | CTGGAAAACCCAAACTTTTCATTA | s | 62 |
|  |  | GCCAGGAAAAGTAACCAGGTGC | as |  |
| *CASPASE9* | NM_001205504.2 | CGACGCTTCCACCTGCTG | s | 65 |
|  |  | CACAATTCTCTCGACGGACACAG | as |  |
| *CDX2* | NM_001206299.1 | AACCTGTGCGAGTGGA | s | *60* |
|  |  | GGCGTGGACAGTGGTCATAA | as |  |
| *RPL15* | NM_001077866.1 | CACAAGTTCCACCACACTATTGG | s | *61* |
|  |  | TGGAGAGTATTGCGCCTTCTC | as |  |
| *SDHA* | NM_174178.2 | GCAGAACCTGATGCTTTGTG | s | 64 |
|  |  | CGTAGGAGAGCGTGTGCTT | as |  |
| *SOX2* | NM_001105463 | CCATGCAGGTTGACATCGT | s | 60 |
|  |  | ACACAACTACGGAAACTAAAAGTGG | as |  |
| *YWHAZ* | XM_025001429.1 | GCATCCCACAGACTATTTCC | s | 56 |
|  |  | GCAAAGACAATGACAGACCA | as |  |

**Supplementary Table 2.** List of mechanically hatched blastocyst and blastocyst percentage from day 7 to day 9 embryo culture. Percentage of blastocyst with cell fragments (CF+) and blastocyst without cell fragments (CF+) from mechanically hatched blastocysts. Total blastocyst percentage was calculated from total matured oocytes. ‘BL’= blastocyst, ‘N’ = number and ‘%’ = percentage.

|  | Day 7 BL | | |  | Day 8 BL | | |  | Day 9 BL | | | Total matured oocyte |
| --- | --- | --- | --- | --- | --- | --- | --- | --- | --- | --- | --- | --- |
|  | CF+ | CF- | Total BL |  | CF+ | CF- | Total BL |  | CF+ | CF- | Total BL |  |
|  | N (%) | N (%) | N (%) |  | N (%) | N (%) | N (%) |  | N (%) | N (%) | N (%) |  |
| Replicate 1 | 21 (48.8) | 22 (51.2) | 127 (22.1) |  | 32 (74.4) | 11 (25.6) | 176 (30.6) |  | 24 (54.5) | 20 (45.5) | 211 (36.7) | 575 |
| Replicate 2 | 18 (40.0) | 27 (60.0) | 163 (26.1) |  | 16 (34.8) | 30 (65.2) | 209 (33.4) |  | 23 (45.1) | 28 (54.9) | 230 (36.8) | 625 |
| Replicate 3 | 20 (38.5) | 32 (61.5) | 183 (24.3) |  | 26 (39.4) | 40 (60.6) | 264 (35.1) |  | 26 (49.1) | 27 (50.9) | 273 (36.3) | 752 |

**Supplementary Table 3.** List of DAPI positive cells, TUNEL positive cells (#) and percentage of TUNEL positive cells (%) in extruded cells / cell fragments (CF) in zonae pellucidae in day 9 blastocysts.

|  | DAPI positives | #TUNEL positives | %TUNEL positives |
| --- | --- | --- | --- |
| CF in zona-1 | 3 | 2 | 66.7 |
| CF in zona-2 | 2 | 0 | 0 |
| CF in zona-3 | 5 | 1 | 20 |
| CF in zona-4 | 2 | 2 | 100 |
| CF in zona-5 | 2 | 2 | 100 |
| CF in zona-6 | 1 | 1 | 100 |
| CF in zona-7 | 5 | 3 | 60 |
| CF in zona-8 | 9 | 6 | 66.7 |
| CF in zona-9 | 2 | 1 | 50 |
| CF in zona-10 | 0 | 0 | 0 |
| CF in zona-11 | 0 | 0 | 0 |

**Supplementary Table 4.** List of number of blastocyst and zona with cell fragments examined and analyzed in each assay. ‘IF’= immunofluorescence, ‘BL (CF+)’ = blastocyst with cell fragments, ‘BL (CF-)’ = blastocyst without cell fragments, ‘A (E)’ = analyzed (examined), ‘zona’ = cell fragments in zonae pellucidae. ‘D’ = day.

| Assay | qRT-PCR | |  | IF (CDX2+TUNEL) | |  | IF (TUNEL) |  | IF (CDX2+  Phalloidin) |  | qRT-PCR | |
| --- | --- | --- | --- | --- | --- | --- | --- | --- | --- | --- | --- | --- |
| Sample | D8 BL (CF+) | D8 BL (CF-) |  | D8 BL (CF+) | D8 BL (CF-) |  | D9 zona |  | D9 zona |  | D9 zona | D9 BL |
| Replicate 1 | 22 | 23 |  | 5 | 4 |  | 4 |  | 2 |  | 47 | 28 |
| Replicate 2 | 21 | 21 |  | 4 | 5 |  | 5 |  | 5 |  | 55 | 32 |
| Replicate 3 | 21 | 22 |  | 4 | 3 |  | 2 |  | 3 |  | 49 | 27 |

**Supplementary Table 5.** List of CDX2 positive cells and TUNEL positive cells counting in day 8 blastocyst with and without cell fragments (CF) in zonae pellucidae.

‘CDX2+’ = CDX2 positive cells, ‘TUNEL+’ = TUNEL positive cells, ‘BL (CF+)’ = blastocyst with cell fragments, ‘BL (CF-)’ = blastocyst without cell fragments, ICM = inner cell mass, ‘D’ = day.

|  | CDX2+ in total cell | |  | TUNEL+ in total cell | |  | TUNEL+ in ICM | | ICM number | Total cell number |
| --- | --- | --- | --- | --- | --- | --- | --- | --- | --- | --- |
|  | N | % |  | N | % |  | N | % |  |  |
| D8 BL (CF+)-1 | 62 | 68.9 |  | 3 | 3.3 |  | 1 | 3.6 | 28 | 90 |
| D8 BL (CF+)-2 | 23 | 44.2 |  | 15 | 28.8 |  | 7 | 24.1 | 29 | 52 |
| D8 BL (CF+)-3 | 79 | 81.4 |  | 0 | 0.0 |  | 0 | 0.0 | 18 | 97 |
| D8 BL (CF+)-4 | 47 | 64.4 |  | 6 | 8.2 |  | 4 | 15.4 | 26 | 73 |
| D8 BL (CF+)-5 | 52 | 74.3 |  | 0 | 0.0 |  | 0 | 0.0 | 18 | 70 |
| D8 BL (CF+)-6 | 45 | 72.6 |  | 2 | 3.2 |  | 0 | 0.0 | 17 | 62 |
| D8 BL (CF+)-7 | 60 | 64.5 |  | 2 | 2.2 |  | 1 | 3.0 | 33 | 93 |
| D8 BL (CF+)-8 | 33 | 49.3 |  | 1 | 1.5 |  | 0 | 0.0 | 34 | 67 |
| D8 BL (CF+)-9 | 122 | 63.9 |  | 8 | 4.2 |  | 4 | 5.8 | 69 | 191 |
| D8 BL (CF+)-10 | 57 | 79.2 |  | 0 | 0.0 |  | 0 | 0.0 | 15 | 72 |
| D8 BL (CF+)-11 | 98 | 70.5 |  | 2 | 1.4 |  | 2 | 4.9 | 41 | 139 |
| D8 BL (CF+)-12 | 97 | 56.7 |  | 6 | 3.5 |  | 2 | 2.7 | 74 | 171 |
| D8 BL (CF+)-13 | 51 | 49.5 |  | 2 | 1.9 |  | 0 | 0.0 | 52 | 103 |
|  | CDX2+ in total cell | |  | TUNEL+ in total cell | |  | TUNEL+ in ICM | | ICM number | Total cell number |
|  | N | % |  | N | % |  | N | % |  |  |
| D8 BL (CF-)-1 | 77 | 52.4 |  | 2 | 1.4 |  | 1 | 1.4 | 70 | 147 |
| D8 BL (CF-)-2 | 44 | 61.1 |  | 4 | 5.6 |  | 1 | 3.6 | 28 | 72 |
| D8 BL (CF-)-3 | 44 | 52.4 |  | 8 | 9.5 |  | 2 | 5.0 | 40 | 84 |
| D8 BL (CF-)-4 | 63 | 51.2 |  | 9 | 7.3 |  | 2 | 3.3 | 60 | 123 |
| D8 BL (CF-)-5 | 58 | 74.4 |  | 2 | 2.6 |  | 1 | 5.0 | 20 | 78 |
| D8 BL (CF-)-6 | 69 | 65.1 |  | 3 | 2.8 |  | 2 | 5.4 | 37 | 106 |
| D8 BL (CF-)-7 | 93 | 78.8 |  | 0 | 0.0 |  | 0 | 0.0 | 25 | 118 |
| D8 BL (CF-)-8 | 87 | 79.8 |  | 1 | 0.9 |  | 1 | 4.5 | 22 | 109 |
| D8 BL (CF-)-9 | 68 | 85.0 |  | 1 | 1.3 |  | 1 | 8.3 | 12 | 80 |
| D8 BL (CF-)-10 | 75 | 67.6 |  | 7 | 6.3 |  | 5 | 13.9 | 36 | 111 |
| D8 BL (CF-)-11 | 78 | 71.6 |  | 3 | 2.8 |  | 3 | 9.7 | 31 | 109 |
| D8 BL (CF-)-12 | 63 | 64.3 |  | 3 | 3.1 |  | 2 | 5.7 | 35 | 98 |
